# Immunomodulatory and Regenerative Effects of Mesenchymal Stem Cell-Derived Extracellular Vesicles in Renal Diseases

**DOI:** 10.3390/ijms21030756

**Published:** 2020-01-23

**Authors:** Kenji Tsuji, Shinji Kitamura, Jun Wada

**Affiliations:** Department of Nephrology, Rheumatology, Endocrinology and Metabolism, Okayama University Graduate School of Medicine, Dentistry, and Pharmaceutical Sciences, 2-5-1 Shikata-cho, Okayama 700-8558, Japan; gmd422036@s.okayama-u.ac.jp (K.T.); junwada@okayama-u.ac.jp (J.W.)

**Keywords:** mesenchymal stem cell, extracellular vesicles, microRNA, immunomodulation, renal regeneration, acute kidney injury, chronic kidney disease

## Abstract

Mesenchymal stem cells (MSCs) have immunomodulatory and regenerative effects in many organs, including the kidney. Emerging evidence has shown that the trophic effects from MSCs are mainly mediated by the paracrine mechanism rather than the direct differentiation of MSCs into injured tissues. These secretomes from MSCs include cytokines, growth factors, chemokines and extracellular vesicles (EVs) containing microRNAs, mRNAs, and proteins. Many research studies have revealed that secretomes from MSCs have potential to ameliorate renal injury in renal disease models, including acute kidney injury and chronic kidney disease through a variety of mechanisms. These trophic mechanisms include immunomodulatory and regenerative effects. In addition, accumulating evidence has uncovered the specific factors and therapeutic mechanisms in MSC-derived EVs. In this article, we summarize the recent advances of immunomodulatory and regenerative effects of EVs from MSCs, especially focusing on the microRNAs.

## 1. Introduction

Mesenchymal stem cells (MSCs) are multipotent cells with the abilities of self-renewal, regeneration, and multilineage differentiation with respect to tissues such as bone, muscle, and adipocyte [1]. MSCs can be isolated from different kinds of tissues, including bone marrow, adipose tissue, umbilical cord, amniotic fluid, and placenta [1]. MSCs have been shown to ameliorate renal injury in a number of kidney disease models, including acute kidney injury (AKI), chronic kidney disease (CKD), and diabetic nephropathy. MSCs have been shown to directly differentiate and replace the injured area under tissue injury. In addition, MSCs secrete trophic factors such as cytokines, chemokines, growth factors, and extracellular vesicles (EVs) containing proteins, mRNA, and microRNAs (miRNAs). Accumulating evidence has demonstrated that the paracrine action is the dominant mechanism for ameliorating kidney injury. These therapeutic mechanisms are mediated through many biological processes, including anti-apoptosis, anti-inflammation, angiogenesis, and antifibrosis. Among the secretomes from MSCs, EVs have been shown to be the important mediator for renal protection in MSC therapy [2]. Accumulating evidence has revealed the specific miRNAs and proteins in MSC-derived EVs in renal diseases. In this review article, we summarize the current knowledge about therapeutic potential of EVs from MSCs, especially focusing on the immunomodulatory and regenerative effects.

## 2. Extracellular Vesicles

EVs are microvesicles enclosed by membrane from cells, and they deliver various biological molecules, including miRNAs, mRNA, long non-coding RNAs, tRNAs, circular RNAs, rRNAs, and proteins, into recipient cells [3], and influence their biological functions. EVs have been shown to have key roles in cell–cell communication in health and disease. With the ability to deliver a variety of factors, EVs have been shown to regulate a variety of biological functions, including cell proliferation, anti-apoptosis, antifibrosis, anti-inflammation, and angiogenesis. In fact, EVs from MSCs have been shown to mediate therapeutic effects in renal diseases through immunomodulation and regeneration. EVs can be grouped into several categories, including exosomes, microvesicles, and apoptotic bodies [4]. The diameters of exosomes are between 30 and 100 nm; microvesicles are 50–1000 nm, and apoptotic bodies are 1–5 mm. EVs can also be isolated from urine, amniotic fluid, plasma, and serum, and have been reported to be the biomarkers for specific diseases [5]. EVs have the advantage that they are able to internalize into target cells directly and quickly [6]. Many researchers demonstrated that the treatment of MSC-derived EVs ameliorated renal injury in rodent AKI and CKD models. There was the important problem of heterogeneity because of the different protocols of isolation and characterization. Therefore, the International Society of Extracellular Vesicles (ISEV) was instituted in 2011 to uniform the nomenclatures and methodologies for the isolation and characterization of EVs.

## 3. Proteins of MSC-Derived EVs

MSCs secrete many varieties of cytokines, chemokines, and growth factors. In addition, MSC-derived EVs also deliver these factors to recipient cells directly. Several studies conducted the proteomic analysis of MSC-derived EVs. For example, Mardpur et al. reported 938 proteins by quantitative proteomic technique [7]. They used the Kyoto Encyclopedia of Genes and Genomics (KEGG) and indicated that enriched proteins in MSC-EVs are involved in extracellular matrix (ECM) receptor interaction, focal adhesion, phosphatidylinositol 3-kinase (PI3K)-protein kinase B (AKT) signaling, actin cytoskeleton, and biosynthesis of antibiotics. They also performed the analyses using Protein Analysis Through Evolutionary Relationships (PARTNER) analysis software, clustered MSC-EVs-proteins into 11 biological processes, and indicated that the cellular and metabolic processes are the highest while immune system response and response to stimulus are also involved. In addition, they revealed that MSC-EVs contain hepatocyte growth factor (HGF), Interleukin (IL)-10, Leukemia inhibitory factor (LIF), C-C motif chemokine ligand (CCL)-2 (also known as monocyte chemotactic protein-1 (MCP-1)), Vascular endothelial growth factor (VEGF)C, CCL-20, C-X-C motif chemokine ligand (CXCL)-2, CXCL-8, and CXCL-16. Among these factors, HGF has been shown to protect from renal injury in many experimental reports. For example, HGF has been shown to inhibit macrophage infiltration by inhibiting CCL-2 in diabetic rat kidney [8]. HGF has also been shown to inhibit glomerulosclerosis through the suppression of transforming growth factor (TGF)-β in diabetic rat kidney [8]. In addition, HGF and TNF-stimulated gene (TSG)-6 from MSCs have been shown to ameliorate inflammation in albumin-induced CKD model via the down-regulation of IL-6, IL-8, TNF-α, CCL-2, and CCL-5, which was partly mediated by the regulation of nuclear factor kappa-light-chain-enhancer of activated B cells (NF-κB) signaling [9]. Kim et al. also reported the proteomic analysis of human bone marrow MSC-EVs by the Database for Annotation, Visualization and Integrated Discovery (DAVID) software and indicated that MSC-EVs contain 730 proteins, and these proteins are mainly involved in cell cycle, proliferation, migration, adhesion (fibronectin, ezrin, Ras GTPase-activating-like protein IQGAP1, and integrin), signaling molecules (mitogen-activated protein kinase (MAPK)1 and CDC42) and morphogenesis [10]. They also indicated these proteins include growth factor receptor (platelet-derived growth factor receptor (PDGFR), epidermal growth factor receptor (EGFR), and urokinase-type plasminogen activator receptor (uPAR)), Wnt, TGF-β, MAPK, peroxisome proliferator-activated receptor (PPAR), and bone morphogenetic protein (BMP) signaling. This broad spectrum of cellular processes and molecular functions suggest a variety of trophic mechanisms. Eirin et al. also reported the proteomic analysis using porcine adipose tissue-derived MSC-EVs. They indicated enriched proteins in MSC-EVs are involved in angiogenesis, blood coagulation, ECM remodeling, inflammatory response, and apoptosis [11]. They indicated that MSC-EVs contain key proteins for angiogenesis, such as VEGF, angiopoietin-related protein-4, platelet-derived growth factor-C (PDGFC), and WNT7B. Anderson et al. also reported 1927 proteins in MSC-EVs, revealing the modulation of angiogenesis via NF-κB signaling [12]. Lai et al. also performed the proteome analysis of MSC-EVs and indicated the 20S proteasome as the candidate to ameliorate tissue damage [13]. In addition, the specific factor for renal protection has also been reported. Jia et al. demonstrated that 14-3-3ζ in MSC-EVs protected from cisplatin-induced renal injury through the promotion of autophagy by the interaction with autophagy-related gene (ATG)-16L [14]. Taken together, MSC-EVs-derived proteins might have important roles in tissue protection through a variety of mechanisms.

## 4. mRNAs of MSC-Derived EVs

MSC-EVs contain a variety of mRNAs, and these mRNAs can be transferred into recipient cells. Valadi et al. demonstrated that these transferred mRNA did translate into proteins that can be functional in the recipient cells [15]. In addition, Batagov et al. reported that exosomal mRNAs have a specific structural pattern with the larger fraction in the secreted RNAs as the fragment localizes to the transcript closer to the 3′-end, implying that exosomal RNAs might act as competing RNAs to regulate stability, localization, and mRNA translation [16]. Accumulating evidence indicated the mRNA profile in MSC-EVs. For example, Bruno et al. reported, using reverse transcription (RT)-polymerase chain reaction (PCR) arrays, that these mRNAs might be involved in a variety of biological functions, including immune regulation, cell cycle, actin cytoskeleton regulation, and ECM remodeling [17]. Eirin et al. also characterized the RNA cargos in MSC-EVs, indicating that these mRNAs might be involved in angiogenesis, adipogenesis, transcription, and TGF-β signaling [18]. Importantly, regenerative effects of MSC-EVs treatment in gentamycin and glycerol-induced AKI models were abolished by RNA inactivation with RNase treatment, indicating the important roles of mRNAs and/or miRNAs on renal regeneration [19,20].

## 5. miRNAs of MSC-Derived EVs

miRNAs are noncoding, single-stranded, small-molecule RNAs. miRNAs silence their target genes through binding to the 3′-UTR. miRNAs in MSC-EVs have been reported to ameliorate renal injury in AKI and CKD rodent models. miRNAs are thought to be one of the essential contributors in MSC-EVs treatment. Indeed, the regenerative effects of MSC-EVs on renal injury were abolished with the knockdown of the *ribonuclease III Drosha* gene, which is essential for the initial processing step of miRNAs [19], suggesting the dominant roles of miRNAs for renal protection. Several reports indicated that MSC-EVs contain a variety of miRNAs, although the heterogeneity of factors in the different origins of MSCs has also been indicated [21]. The detailed miRNA analysis in MSC-EVs and the possible heterogeneity have previously been reviewed [21]. Previous reports indicated that RNase treatment into MSC-EVs abolished the trophic effects in ischemia/reperfusion (I/R)-induced AKI animal models [17,22], indicating the importance of mRNA and/or miRNAs for renal protection. In addition, accumulating evidence has gradually uncovered the therapeutic mechanisms mediated by specific trophic miRNAs in MSC-EVs for renal immunomodulation and regeneration in AKI and CKD animal models in vivo and in vitro (Table 1). For example, Collino et al. reported the regenerative effects of MSC-EVs-derived miRNAs in glycerol-induced AKI rat model [19]. They indicated that regenerative potential was abolished by the knockdown of the *ribonuclease III Drosha* gene, which corresponded to the reduced expression of miR-483-5p, miR-191, miR-28-3p, miR-423-5p, miR-744, miR-129-3p, miR-24, and miR-148a family, indicating the important roles of these miRNAs for renal regeneration. In the same line, Zou et al. reported the protective effects of MSC-EVs in I/R-induced AKI rat model and revealed that MSC-EVs promoted cell proliferation and inhibited renal fibrosis through the C-X3-C motif chemokine ligand (CX3CL)1 regulated by miR-15a, miR-15b, and miR-16 [23]. Gu et al. reported that miR-30 in MSC-EVs preserved mitochondrial morphology and inhibited tubular cell apoptosis in I/R-induced AKI rat model [24]. Zhu et al. reported that miR-199a-3p in MSC-EVs inhibited semaphorin3a (Sema3A) expression, which might activate AKT and extracellular signal-regulated kinase (ERK)-signaling pathway, thereby ameliorating cell apoptosis [25]. In addition to the trophic effects in AKI injury models, the renal protection in CKD animal models has been reported. Wang et al. demonstrated that the overexpression of let-7c in MSC-EVs ameliorated renal fibrosis in unilateral ureteral obstruction (UUO) model [26]. In vitro analysis also revealed that MSC-EVs blocked TGF-β-mediated epithelial-mesenchymal transition (EMT) in renal proximal tubular epithelial cells [27,28], and these effects were partly through miR-133b-3p and miR-294 [27]. They also reported that let-7c transferred from MSC-EVs decreased the expression of collagen type IV and matrix metalloproteinase-9 (MMP-9) via inhibition of TGF-β1 signaling in UUO model [26]. In the same line, He et al. reported MSC-EVs treatment ameliorated UUO-induced renal injury via miR-29, miR-30, and miR-210-3p [28]. Grange et al. applied MSC-EVs treatment in type 1 diabetes model and revealed the amelioration of renal fibrosis mediated by miRNA [29]. They suggested that miRNA-EVs might be involved in TGF-β, epidermal growth factor receptor (EGFR), platelet-derived growth factor receptor (PDGFR), ADP-ribosylation factor 6 (ARF6), mammalian target of rapamycin (mTOR), VEGF, p53-apoptosis, ataxia telangiectasia-mutated (ATM), and tumor necrosis factor (TNF) pathway. Zhong et al. reported that miR-451 in MSC-EVs ameliorated diabetic kidney injury through the inhibition of EMT, and the effect relied on the improved cell cycle arrest and the downregulation of P15INK4b (P15) and P19INK4d (P19) [30]. In vitro, Xiang et al. reported that MSC-EVs enhanced autophagy of HK-2 cells and immortalized human proximal tubular cells through miR-145 by inhibiting the PI3K/AKT/mTOR signaling pathway, suggesting a therapeutic effect in renal diseases. These multiple therapeutic mechanisms might be mediated by the combination of several miRNAs in MSC-EVs.

## 6. Versatile MSC-EVs-Derived miRNAs Modulate Renal Injury and Homeostasis

In addition to these miRNAs that have directly been shown to be protective in renal diseases, there is accumulating evidence of miRNA being associated with the renal regeneration and also pathophysiology in renal diseases. Among the miRNAs summarized in the previous review paper indicating the miRNA profile from MSC-EVs [21], miR-1246, -23a, -451a, -125b, -199a, -let-7a, -4454/7975, -21, -let-7b, -100, -29a, -144, -29b, -22, -630, -221, -let-7i, -424, -191, -25, -130a, -376a, -27b, -30, -210, -24, -1202, -638, -148a, -532, -378, -let-7f, 486, -10a, -10b, -222, -143, -199b, -218, -135b, -203, -219, -299, -302b, -145, -338, -1260, and -1908, we searched the related papers and summarized the effects of these miRNAs reported in renal diseases (Table 2).

miR-21: miR-21 is one of the most reported miRNAs in renal injury. miR-21 has been reported to silence phosphatase and tensin homolog deleted from chromosome 10 (PTEN) and glycogen synthase kinase (GSK)-3β, thereby reducing NF-κB activity in macrophage [71]. In renal diseases, Song et al. reported that miR-21 ameliorated I/R-induced AKI by inhibiting tubular cell apoptosis and dendritic cell maturation [33]. On the other hand, miR-21 has been shown to correlate with tubulointerstitial injury in kidney biopsies patients in diabetic patients. Indeed, Koling et al. reported that the inhibition of miR-21 ameliorated renal injury in diabetic mice [34]. Xu et al. described the miR-21 as a double-edged sword because of the divergent pathophysiological processes, including inflammation and angiogenesis, and complex roles both on protective and pathological pathways in renal diseases [72]. They indicated that the preconditioning of upregulation of miR-21 contributed to the renal protection in subsequent I/R-induced renal injury through the anti-apoptosis. miR-21 has been shown to be downstream of AKT that mediates its anti-apoptotic effect through the suppression of Fas ligand [73]. Zhu et al. reported that miR-21 inhibited lipopolysaccharide (LPS)-induced NF-κB activation and IL-6 and enhanced IL-10 expression in monocyte cells in lung injury [74]. Conversely, long-term elevation of miR-21 might promote the progression of renal fibrosis [72]. Indeed, miR-21 promoted angiotensin II-induced renal fibrosis by activating the TGF-β1 pathway via suppressing PRARα [37]. Tang et al. reported that the silencing miR-21 inhibited renal interstitial fibrosis via targeting ERK1/2 signaling in mice [36]. miR-21 has also been shown to promote renal fibrosis by targeting PPARα [35]. Furthermore, miR-21 has also been shown to induce renal fibrosis through multiple pathways including AKT, ERK/MAPK, and it has been shown to target Smad7, which is a negative regulator of TGF-β/Smad3 pathway [38,75]. These complex mechanisms of miRNA might result in the effects of the double-edged sword.

miR-199: As described ahead, miR-199a-3p inhibits Sema3A, which activates AKT and ERK signaling, thereby ameliorating cell apoptosis [25]. In the same line, Sun et al. reported that the inhibition of miR-199a-5p suppressed cyst proliferation and induced cell apoptosis in autosomal dominant polycystic kidney disease (ADPKD) tissue and cells by targeting *CDKN1C* [39]. On the other hand, Yang et al. reported that the inhibition of miR-199a-3p reduced cisplatin-induced cell apoptosis and inhibited caspase-3 activity [40]. mTOR is the target gene of miR-199a [76], regulating cell survival, proliferation, migration, and apoptosis. In addition, miR-199a-3p increased TGF-β1-induced renal fibrosis via silencing of SOCS7 and STAT3 [41]. miR-199a has also been reported to be involved in inflammation via modulating the activation of NF-κB by targeting Klotho in lupus nephritis [42]. Sun et al. reported the increased level of serum miR-199b-5p in diabetic patients [43]. They also indicated that the inhibition of miR-199b ameliorated TGF-β1-induced EMT and renal fibrosis via targeting SIRT1 in diabetic nephropathy [43]. Kang et al. suggested that miR-199b-5p targets klotho, thereby promoting renal tubular injury in diabetic nephropathy [77]. In summary, miR-199 might have dual effects of the double-edged sword in renal diseases similar to miR-21.

let-7 family: the let-7 family has been reported to target collagen I [78,79] as well as being involved in cell cycle, cell proliferation, apoptosis, and immune system. Yan et al. demonstrated that naringenin ameliorated kidney injury through let7a-TGFBR1 signaling in diabetic nephropathy [44]. In addition, the let-7 family has been shown to protect against TGF-β1-induced collagen accumulation, and it is suggested that Col1a2 and Col4a1 might be the targets of the let-7 family [45]. Let-7b has also been reported to regulate renal fibrosis in proximal tubular cells and mesangial cells via regulation of TGFBR1 under renal injury [46]. In addition, let-7i has been reported to target toll-like receptor (TLR)4, thereby inhibiting LPS-induced inflammatory kidney injury [47]. As described ahead, the transfer of let-7c from MSC-EVs ameliorated renal fibrosis in UUO model through the downregulation of Col4a1, MMP-9, TGF-β1, and TGFBR1 [26]. Taken together, the let-7 family might be the promising target for ameliorating renal fibrosis and inflammation in renal diseases.

miR-30: As described ahead, miR-30 preserved mitochondrial morphology and inhibited cell apoptosis in I/R-induced AKI rat model [24]. miR-30c has been shown to target *Bnip3L* and *Hspa5*, thereby inhibiting cisplatin-induced tubular epithelial cell apoptosis [48]. In addition, He et al. reported that miR-30a is associated with the inhibition of UUO-induced renal fibrosis under MSC-EVs treatment [28]. miR-30a has been reported to target the *snail* in hepatocytes [80]. Zhang et al. indicated that miR-30c-5p might decrease I/R-induced renal injury by promoting M1 macrophage toward M2 macrophage and changes in inflammatory cytokines [49]. In addition, miR-30 might protect podocyte by preventing uPAR-ITGB3 signaling through the calcineurin–NFATC pathway [50]. In the same line, Yang et al. reported that TGF-β induced podocyte injury through the downregulation of miR-30 [81]. Angiotensin II has been shown to induce calcineurin signaling and podocyte injury by reducing miR-30, indicating that miR-30 expression might be protective for podocyte [51]. miR-30e has been shown to target *Bnip3L* and protect against aldosterone-induced podocyte apoptosis and mitochondrial dysfunction [52]. miR-30a has also been shown to inhibit EMT of podocyte through downregulation of NFATc3 [53]. Taken together, miR-30 might have potentials to mediate a variety of trophic effects, including anti-apoptosis, anti-inflammation, and antifibrosis, as well as the protection of podocytes.

miR-29: miR-29a has been reported to target collagen I [78,79] and is known to act as fibrotic regulator in several tissue types [54]. In renal diseases, miR-29a has been reported to protect from diabetic nephropathy via regulation of PPARγ and CB1R [54]. miR-29a has also been shown to modulate DKK1/Wnt/β-catenin signaling, thereby protecting from diabetic glomerular dysfunction [55]. In addition, Wang et al. reported that TGF-β1 reduced the expression of miR29a/b/c family [82]. TGF-β1 inhibited the expression of miR-29 family, thereby promoting fibrosis via increase of ECM component [82]. Hu et al. reported that miR-29b ameliorated angiotensin II-induced EMT of rat tubular epithelial cells through the regulation of PI3K/AKT signaling [56]. In the same line, miR-200, miR-29, and miR-143 have been reported to affect the progression of TGF-β-dependent EMT and fibrosis [83,84]. miR-29 has also been shown to attenuate renal fibrosis in UUO model by downregulating TGF-β signaling [85]. Saito et al. also demonstrated that miR-29b delivery by adeno-associated virus attenuated renal fibrosis in UUO model by suppressing *snail* expression [57]. In summary, miR-29 might have the potential to block the progression of renal fibrosis through the regulation of TGF-β1/EMT axis.

miR-145: As described ahead, miR-145 might promote autophagy of HK-2 cells by inhibiting the PI3K/AKT/mTOR-signaling pathway. In addition, Liu et al. reported that miR-145 ameliorated TGF-β1-induced EMT in renal proximal tubular cells possibly by inhibiting TGF-β-smad signaling pathway [58]. In the same line, Liu et al., reported that miR-145 overexpression inhibited high-glucose-induced EMT and fibrosis via downregulating ZEB2 [59]. Therefore, miR-145 might be the promising target to protect from kidney injury through the regulation of autophagy and EMT.

miR-210: As described ahead, MSC-EVs might ameliorate UUO-induced renal injury via miR-210-3p [28]. miR-210 has been shown to be upregulated in response to hypoxia-inducible factor (HIF). miR-210 has also been reported to be involved in various pathophysiological pathways such as oxidative stress, cancer, and apoptosis [86]. Liu et al. indicated that miR-210 protected renal tubular cells from apoptosis by targeting HIF-1α [60]. In addition, Liu et al. reported that miR-210 promoted angiogenesis by activation of VEGF pathway in I/R-induced kidney injury [61]. Zhang et al. suggested that MSC-EVs treatment in cisplatin-induced AKI model might ameliorate renal inflammation and cell apoptosis via miR-210/*Serpine1* and miR-378/*Fos* axis [62]. Although further analysis is required to confirm these effects, miR-210 might protect from renal injury in multiple mechanisms.

miR-22: Zhang et al. reported that miR-22 promoted renal fibrosis by targeting PTEN/AKT/mTOR pathway and suppressing autophagy in diabetic nephropathy [63]. Indeed, the inhibition of miR-22 ameliorated kidney fibrosis by targeting BMP-6, BMP-7, and BMP type I receptor [64]. BMP-7 is known to have antifibrotic function through the regulation of EMT and synthesis of ECM [87]. BMP-7 from MSCs has been reported to ameliorate glomerular fibrosis through the inhibition of TGF-β signaling in vivo and in vitro [88]. Therefore, miR-22 might be associated with the progression of renal injury.

miR-125b: miR-125b has been shown to be increased by NF-E2-related factor 2 (Nrf2), which results in the inhibition of aryl hydrocarbon receptor (AhR) repressor and protection from cisplatin-induced renal injury via anti-apoptosis [65]. On the other hand, miR-125b has been shown to contribute to high-glucose-induced reactive oxygen species (ROS) generation and renal tubular cell apoptosis by downregulation of angiotensin-converting-enzyme (ACE) [66]. Further analysis is required to explore the effect of miR-125b in renal diseases.

miR-130a: Liang et al. reported that miR-130a protected LPS-induced glomerular injury by Klotho upregulation [67]. They also indicated that miR-130a activated PI3K/AKT pathway and inhibited Wnt and NF-κB pathway [67]. Liu et al. reported that miR-130a-5p prevented angiotensin II-induced podocyte apoptosis by modulating phospholipase A2 receptor (PLA2R) [68]. In spite of these trophic effects, miR-130a-3p has been reported to target SnoN, thereby promoting renal fibrosis via TGF-β1/Smad pathway, similar to miR-23a [69]. Further analysis is still required to conclude the potential effects of miR-130a in renal diseases.

miR-23a: miR-23a has been shown to be increased in renal tissue in patients with type 2 diabetes mellitus (DM) [70]. Xu et al. reported that miR-23a directly targeted SnoN gene, and miR-23a overexpression promoted high glucose-induced EMT and renal fibrogenesis [70], suggesting the pathophysiological role of miR-23a on the progression of renal fibrosis.

Other possible candidates:

In spite of the limited number of reports in renal diseases, there are several candidate miRNAs associated with renal regeneration and/or pathophysiology.

miR-221: Di et al. reported that miR-221 might target Ets-1, thereby preventing angiotensin II-induced renal fibroblast activation and fibrosis [89].

miR-424: Chen et al. reported that miR-424 expression was increased in I/R renal injury and miR-424 attenuated I/R-induced renal injury through the inhibition of apoptosis via death receptor 6 [90].

miR-191: Qin et al. reported that miR-191-5p reduces sepsis-induced acute kidney injury by targeting oxidative-stress-responsive 1 (OXSR1) in rat model [91].

miR-25: Oh et al. reported that miR-25 expression was downregulated under diabetic condition, which resulted in the increase of NADPH oxidase 4 (NOX4) in early diabetic nephropathy [92].

miR-27b: Zheng et al. reported that miR-27b enhanced puromycin aminonucleoside (PAN)-induced apoptosis and cytoskeleton destruction in podocytes [93].

miR-24: Lorenzen et al. reported that the inhibition of miR-24 in I/R-induced renal injury mice reduced tubular cell apoptosis [94]. On the other hand, Liu et al. reported that miR-24-3p inhibited mesangial cell proliferation and fibrosis through the downregulation of fibroblast growth factor (FGF)-11 [95].

miR-486: Vinas et al. reported that miR-486-5p ameliorated I/R-induced kidney injury via inhibiting PTEN and cell apoptosis [96].

miR-10a: Shan et al. reported that HDAC/miR-10a/CREB1 axis might be associated with renal injury in type 2 DM [97].

miR-218: Yang et al. reported that miR-218 promoted high-glucose-induced apoptosis in podocytes through the regulation of heme oxygenase (HO)-1 [98].

miR-135b: Yang et al. reported that miR-135 family (miR-135a and miR-135b) mediated podocyte injury through the activation of Wnt/β-catenin signaling [99].

miR-203: Liu et al. reported that low miR-203 expression might promote diabetic nephropathy via increasing TLR4 [100].

miR-1908: Xie et al. reported that the transfection of miR-1908 inhibited renal fibrosis via targeting TGF-β1 [101].

## 7. Immunomodulatory Effect of EVs from MSCs against Renal Injury

MSCs have been shown to modulate immune cells such as T cells, dendric cells, B cells, natural killer (NK) cells, and regulatory T cells via soluble factors, including indoleamine 2,3 dioxygenase (IDO), nitric oxide (NO), prostaglandin E2 (PGE2), HGF, and TGF-β1, as well as via direct cell–cell contact [102]. In addition, factors in MSC-EVs are capable of modulating immune cells. The modulation of immune system is one of the most important functions of MSC-EVs therapy among all biological processes. For example, Wang et al. reported that MSC-EVs treatment in cisplatin-induced AKI reduced the levels of TNF-α and IL-1β in renal epithelial cells in vitro [103]. In addition, MSC-EVs treatment, like MSCs injection, decreased TNF-α and IL-1β expression in I/R-induced rat renal injury model [104]. In the same line, MSC-EVs treatment prevented gentamicin-induced AKI by downregulating IL-6 and TNF-α as well as up-regulating IL-10 [105]. Wang et al. also reported that MSC-EVs treatment in I/R-induced AKI rat reduced the TNF-α mRNA expression [106]. Eirin et al. reported that MSC-EVs treatment attenuated renal inflammation after renal artery stenosis, and the effect was partly mediated by IL-10 mRNA in MSC-EVs [107]. IL-10 is known to regulate the function of immune cells and determine the M2 macrophage phenotype. M2 macrophage might attenuate inflammation and promote tissue repair. They also demonstrated the decreased expression of TNF-α, IL-6, and IL-1β [107], indicating the complex roles of MSC-EVs on immunomodulation. They also demonstrated that MSC-EVs treatment shifted the renal macrophage phenotype from M1 toward M2. Zou et al. also suggested that NK cell regulatory process is involved in the MSC-EVs effect in I/R-induced AKI [108]. Choi et al. demonstrated that MSC-EVs treatment in UUO model decreased macrophage infiltration in kidney detected by F4/80 staining [109]. Macrophages are the key inflammatory cells involved in kidney inflammation. In addition, He et al. reported that lymphocyte infiltration was reduced with MSC-EVs treatment in 5/6 subtotal nephrectomy mouse model [110]. As described ahead, several specific miRNAs have been reported to possess immunomodulatory effects. For example, miR-21 has been reported to silence PTEN and GSK-3β, thereby reducing NF-κB activity in macrophage [71]. miR-21 inhibited LPS-induced NF-κB activation and IL-6 and enhanced IL-10 expression in monocyte cells [74]. miR-199a has also been reported to be involved in inflammation via modulating the activation of NF-κB by targeting Klotho in Lupus nephritis [42]. miR-210 and miR-378 have also been shown to regulate inflammation by targeting *Serpine1* and *Fos* in cisplatin-induced AKI model [62]. Zou et al. reported that MSC-EVs treatment blocked macrophage infiltration through the downregulation of CX3CL1 by miR-15a, miR-15b, and miR-16 [23]. CX3CL1 has potential as chemo-attraction for macrophage, and thereby the inhibition of CX3CL1 might reduce the accumulation of macrophage in the kidney. As shown ahead, MSC-EVs contain proteins, including HGF, IL-10, LIF, CCL-2, VEGFC, CCL-20, CXCL-2, CXCL-8, CXCL-16, denfensinα1, HERC5, and IFITM2 that are associated with inflammation [7]. CCL2, VEGFC, and CCL20 might bring immune cells nearby and induce immunosuppressive processes. CXCL2, CXCL8, CXCL16, denfensinα1, HERC5, and IFITM2 have been reported to have a role on immune cell chemotaxis and neutrophil degranulation. HGF might inhibit macrophage infiltration by inhibiting CCL-2 in diabetic rat kidney [8] and ameliorate inflammation in albumin-induced CKD model through the downregulation of IL-6, IL-8, TNF-α, CCL-2, and CCL-5 [9]. HGF, IL-10, and TGF-β1 are important factors in suppressing T cell proliferation. TGF-β1 also induces CD4(+)/CD25(+)-regulatory T cells that regulate inflammatory response. NO is also known to have important immunomodulatory effects. NO suppresses T cells through cell cycle arrest and apoptosis. NO also has roles in M1/M2 macrophage fate and cytokine receptor and T cell receptor (TCR) signaling. Gennai et al. reported, using ex vivo perfused human lungs rejected for transplantation, that MSC-EVs increased the NO level in the perfusate [111], suggesting the possibility that MSC-EVs might regulate NO expression and thereby have the potential in immunomodulation under tissue injury. PGE2 is another important factor that affects immunomodulation, especially through the regulation of T cells. PGE2 has been shown to shift Th1 toward Th2/Th17 by inhibiting pro-inflammatory cytokines and inducing Th2 cytokines, including IL-4 and IL-5. Harting et al. demonstrated that MSC-EVs treatment increased the expression of cyclooxygenase-2 (Cox-2) and PGE2, and attenuated pro-inflammatory cytokines [112]. Taken together, MSC-EVs have the strong potential to promote immunomodulation in a variety of mechanisms.

In spite of the trophic effects of MSC-EVs in the point of immunomodulation, MSC-EVs treatment potentially mediates negative immunomodulatory effects [113]. For example, Kilpinen et al. analyzed the secretomes of umbilical-cord-derived MSC-EVs with or without the interferon-gamma (IFN-γ) stimulus and indicated that EVs from MSCs treated with IFN-γ for 24 h contained major histocompatibility Class I (MHCI) and both α and β units of the proteasome complex required for the antigen presentation and activation of T cells [114]. In addition, EVs from MSCs treated with IFN-γ for 48 h also contained major histocompatibility Class II (MHCII). The presence of MHCI and MHCII on the surface of MSC-EVs might trigger the innate immune response. Therefore, MSC-EVs after IFN-γ stimulus might act as immunogenicity and accelerate inflammation. We need to keep in mind the possible negative effects under the MSC-EVs treatment.

## 8. Regenerative Effect of EVs from MSCs against Renal Injury

**Figure 1:**
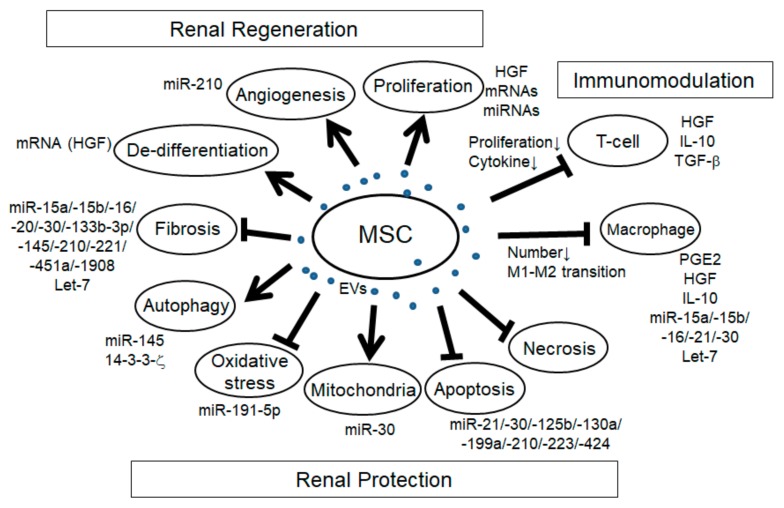
Schema of the effects of mesenchymal stem cell extracellular vesicles (MSC-EVs) in renal injury. Arrows: promotion of the processes; T-bars: inhibition of the processes.

In addition to the immunomodulatory effects, MSC-EVs have the potential to promote renal regeneration and protection in a variety of mechanisms (Figure 1).

### 8.1. Renal Regeneration

In many reports of AKI models induced by I/R and glycerol and cisplatin-induced rodent models, the increase of tubular proliferation was observed under the treatment of MSC-EVs [17,23,105,115,116,117,118,119,120], indicating the regenerative effect of MSC-EVs. Although it is not easy to identify the specific factors for cell proliferation, several factors might be involved. For example, HGF has been reported to enhance mitogenesis in I/R-AKI rat model [121]. mRNA and/or miRNA in MSC-EVs might be involved as well since the enhancement of tubular proliferation with MSC-EVs treatment in glycerol-induced AKI was abolished with RNase treatment. Angiogenesis is another mechanism of renal regeneration in rodent AKI models. MSC-EVs treatment in I/R-induced AKI rat model showed the increase of VEGF expression and capillary vessel density that was abolished with RNase treatment [116]. In addition, MSC-EVs treatment in I/R-induced AKI rat model showed the increase of VEGF and VEGFR2 expression as well as the increase of miR-210, and the overexpression of miR-210 in HUVEC-12 cells increased VEGF and VEGFR2 expression and promoted angiogenesis in vitro, indicating that miR-210 might be involved in MSC-EV-induced angiogenesis by targeting VEGF signaling [61]. The expression of angiogenesis indicators, CD31, von Willebrand factor (vWF), and angiopoietin was upregulated with MCS-EVs treatment in I/R-induced AKI rat model [104]. In addition, in the UUO-induced CKD mouse model, MSC-EVs treatment improved the rarefaction of peritubular capillaries detected by CD31 staining [109]. During renal regeneration after AKI, epithelial de-differentiation has been reported to be an important process that promotes cell survival, migration, and proliferation [122]. HGF, TGF-β1, IGF, and EGF have been reported to be involved in the de-differentiation [122,123]. Indeed, Ju et al. reported that MSC-EVs treatment in I/R-induced AKI rat model promoted tubular epithelial cell de-differentiation via HGF induction that was blocked with RNase treatment, suggesting that HGF induction might be mediated by mRNAs and/or miRNAs [124]. Furthermore, they revealed that the HGF mRNA from MSC-EVs entered the injured tubular cells and was translated into HGF protein, indicating foreign HGF synthesis. In summary, MSC-EVs have a variety of trophic mechanisms for renal regeneration through the regulation of cell proliferation, angiogenesis, and tubular cell de-differentiation.

### 8.2. Renal Protection

The mechanism regulating cell apoptosis is one of the most important aspects of renal protection. Indeed, the anti-apoptotic pathway has been reported in many studies with MSC-EVs treatment in a variety of rodent renal injury models, including AKI induced by I/R [24,31,33,104,106,116,117,118,120,124,125], cisplatin [48,119,126], gentamicin [105], and glycerol [17], hypoxia-induced renal injury [60], aldosterone-induced renal injury [52], and UUO-induced CKD model [109]. Recent advances have gradually uncovered the specific miRNAs, their targets and signaling pathways involved in the effect of anti-apoptosis (Table 2), including PTEN, AKT, mTOR, *dynamin-related protein 1* (*DRP1*), Sema3A, and ERK signaling pathway. Anti-necrosis with MSC-EVs treatment has also been reported in drug-induced AKI, such as with glycerol, cisplatin, and gentamicin [105,115,119]. Autophagy regulation is another aspect of trophic mechanism with MSC-EVs treatment for renal protection. In cisplatin-induced AKI model, the improvement of autophagy was observed with MSC-EVs treatment [14]. In the study, there was an increase of autophagy marker and LC3II expression in vivo and in vitro with MSC-EVs treatment, and it was associated with 14-3-3ζ expression, which regulates ATG-16L. Another group also indicated that MSC-EVs treatment increased the LC3B expression as well as the ATG-5 and ATG-7 gene expressions in normal rat kidney-52E (NRK-52E) cells through the inhibition of mTOR signaling [103]. In addition, Xiang et al. indicated that MSC-EVs enhanced the expression of LC3II and beclin 1 in HK-2 cells through miR-145 targeting PI3K/AKT/mTOR-signaling pathway [32]. Under pathological conditions, autophagy is induced as the adaptive and protective mechanism for cell survival, thus the regulation of autophagy is an important mechanism for renal protection. Preservation of mitochondria is also an important mechanism for renal protection from AKI. *DRP1* is known as the key regulator for mitochondrial fission [127] and is rapidly activated after AKI [128]. Inhibition of *DRP1* has been reported to protect the kidney from AKI injury caused by I/R and cisplatin [128,129]. Indeed, MSC-EVs treatment preserved mitochondrial function via transferring miR-30 that inhibited the expression of *DRP1* [24]. Improvement of oxidative stress with MSC-EVs treatment is also an important mechanism for renal protection against cisplatin and I/R-induced AKI [104,117,119,125]. Oxidative stress via reactive ROS production induces apoptosis, necrosis, and inflammation in AKI. Zhang et al. reported that MSC-EVs treatment alleviated oxidative stress detected by the reduction of malondialdehyde (MDA) and 8-hydroxy-2′-deoxyguanosine (8-OhdG) as well as the enhancement of Nrf2 and HO-1 in I/R-induced AKI rat model [125]. NOX-2, known as the inducer of ROS production, declined with MSC-EVs treatment in I/R-induced AKI rat model [117]. In addition, Zhou et al. reported that MSC-EVs treatment ameliorated oxidative stress in cisplatin-induced AKI detected by the reduction of 8-OhdG-positive cells, which reduced the cell apoptosis [119]. HGF has also been reported to improve oxidative stress through the suppression of GLUT1 [130]. Taken together, several factors in MSC-EVs might be involved in the regulation of oxidative stress. Renal fibrosis is the pathological process of CKD, which is strongly associated with the progression of renal dysfunction. The improvement of renal fibrosis with MSC-EVs treatment was reported in a variety of renal injury models, including I/R-induced AKI rodent model [23,104,116,117,124], renovascular stenosis-induced pig CKD model [107], UUO-induced renal fibrosis model [26,28,109], diabetes nephropathy mouse model [29,30], and 5/6 subtotal nephrectomy mouse model [110]. In vitro experiment using HK-2 cells, MSC-EVs ameliorated TGF-β1-induced EMT, which was mediated by miR-133b-3p and miR-294 [27]. As described ahead, let-7c from MSC-EVs ameliorated renal fibrosis in UUO model with the downregulation of Col4a1, MMP-9, TGF-β1, and TGFBR1 [26]. In addition, miR-451 in MSC-EVs ameliorated renal fibrosis by targeting P15 and P19, thus inhibiting EMT in diabetic model [30]. MiR-29b in MSC-EVs might target *snail*, thereby regulating EMT [57]. Taken together, several MSC-EVs-derived miRNAs have the potential to ameliorate renal fibrosis, mainly through the regulation of TGF-β1-EMT axis. In summary, EV-MSCs have a variety of factors involved in many biological processes for renal generation and protection as well as immunomodulation.

## 9. Conclusions

In this review article, we summarized the recent advances of the immunomodulatory and regenerative effects of MSC-EVs in renal diseases. MSC-EVs treatments have several advantages over MSCs therapy in the point of easy transfer into recipient cells, no risk of tumor formation, and low immunogenicity because of the cell-free sources. Although these cell-free therapies might be promising to treat against renal injury, there are still challenges for clinical use. The heterogeneity of secretomes of MSCs obtained from different tissues might strongly affect the therapeutic potential. In addition, some preconditioning stimuli might affect the quality and quantity of trophic factors in MSC-EVs as well as the engineering or genetic modification for overexpression of specific proteins and miRNAs. Other important challenges are their safety, analysis of MSC cell sources, and isolation method to acquire more effective functions. In conclusion, MSC-EVs therapy might be the promising cell-free therapeutic option for the treatment against renal injury including AKI and CKD. Further exploration is still required for future clinical use for patients with renal diseases.

## Figures and Tables

**Table 1:** The study of miRNA therapy from MSC-EVs in renal diseases.

Animal	Model	MiRNAs	Target Protein/Gene/Signal	Function	Ref.
Rat	Glycerol	miRNAs	multiple	anti-inflammation	[19]
Mouse	I/R	miR-223	NLRP3	anti-apoptosis	[31]
Rat	I/R	miR-15a/-15b/-16	CX3CL1	antifibrosis/anti-inflammation	[23]
Rat	I/R	miR-30	*DRP1*	anti-apoptosis/preserve mitochondria	[24]
HK-2 cell	-	miR-145	PI3K/AKT/mTOR	promote autophagy	[32]
HK-2 cell	-	miR-133b-3p/-294	TGF-β1/EMT	antifibrosis	[27]
Mouse	UUO	miRNAs	EMT	antifibrosis	[28]
Mouse	UUO	let-7c	-	antifibrosis	[26]
Mouse	Diabetes	miR-451a	P15/P19/EMT	antifibrosis	[30]
Mouse	Diabetes	miRNAs	-	antifibrosis	[29]

I/R: Ischemia/reperfusion; UUO: Unilateral ureteral obstruction; CX3CL1: C-X3-C motif chemokine ligand 1; *DRP1*: Dynamic related protein 1; PI3K: Phosphatidylinositol 3-kinase; AKT: Protein kinase B; mTOR: Mammalian target of rapamycin; TGF-β1: Transforming growth factor -β1; EMT: Epithelial-mesenchymal transition; P15: P15INK4b; P19: P19INK4d.

**Table 2:** MiRNAs in MSC-EVs in renal regeneration and pathophysiology.

MiRNA	Renal Injury	Target Protein/Gene/Signal	Function	Reference
miR-21	I/R	PTEN/AKT/mTOR/HIF	anti-apoptosis	-
		PDCD4/NF-κB	inhibit dendritic cell maturation	[33]
	Diabetes	Cdc25/Cdk6	promote DKD:mesangial expansion, renal fibrosis, macrophage	[34]
	UUO	PRARα	promote renal fibrosis	[35]
	UUO	ERK1/ERK2	promote renal fibrosis	[36]
	Ang-II	PRARα/TGF-β1	promote renal fibrosis	[37]
	Diabetes	Smad7/TGF-β/NF-κB	promote renal fibrosis/inflammation	[38]
miR-199				
miR-199a	I/R	Sema3A/AKT/ERK	anti-apoptosis	[25]
	PKD	*CDKN1C*	inhibition of cyst growth	[39]
	Cisplatin	Caspase-3	promote apoptosis	[40]
	TGF-β1	SOCS7/STAT3	promote renal fibrosis	[41]
	Lupus	Klotho/NF-κB	inflammation	[42]
miR-199b	Diabetes	SIRT1	promote renal fibrosis	[43]
Let-7 family	Diabetes	TGFBR1	ameliorate DKD	[44]
	TGF-β1	COL1a2/COL4a1	improve renal injury	[45]
	TGF-β1	TGFBR1	antifibrosis	[46]
	LPS	TLR4	reduce inflammatory injury	[47]
	UUO	MMP9/TGF-β1/TGFBR1	antifibrosis	[26]
miR-30	I/R	*DRP1*	anti-apoptosis/preserve mitochondria	[24]
	Cisplatin	*Bnip3L*/*Hspa5*	anti-apoptosis	[48]
	I/R	M1-M2 macrophage transition	anti-inflammation	[49]
	UUO	EMT	antifibrosis	[28]
	miR-30-KD	uPAR-ITGB3	protect podocytophaty	[50]
	Ang-II	Calcium/calcineurin signaling	protect podocytophaty	[51]
	Aldosterone	*Bnip3*	anti-apoptosis/preserve mitochondria	[52]
	Adriamycin	NFATc3/EMT	protect podocytopathy	[53]
miR-29				
miR-29a	Diabetes	PPARγ/CB1R	ameliorate DKD	[54]
	Diabetes	DKK1/Wnt/bcatenin	ameliorate DKD	[55]
miR-29b	Ang-II	PI3K/AKT	reduce EMT	[56]
	UUO	*Snail*	antifibrosis	[57]
miR-145	-	PI3K/AKT/mTOR	promote autophagy	[32]
	TGF-β1	TGF-β/Smad signaling	antifibrosis	[58]
	High-glucose	ZEB2/EMT	antifibrosis	[59]
miR-210	UUO	EMT	antifibrosis	[28]
	Hypoxia	HIF-1α	anti-apoptosis	[60]
	I/R	VEGF pathway	promote angiogenesis	[61]
	Cisplatin	*Serpine1*	inflammatory regulation	[62]
miR-22	Diabetes	PTEN/AKT/mTOR	promote fibrosis/suppress autophagy	[63]
	UUO	BMP-6/BMP-7 signaling	promote fibrosis	[64]
miR-125b	Cisplatin	AhR receptor	anti-apoptosis	[65]
	High-glucose	ACE/ROS	promote apoptosis	[66]
miR-130a	LPS	Klotho/PI3K/AKT/Wnt/NF-κB	protect glomerular injury	[67]
	Ang-II	PLA2R	anti-apoptosis	[68]
	TGF-β1	SnoN/TGF-β1/Smad	promote fibrosis	[69]
miR-23a	High-glucose	EMT	promote fibrosis	[70]
miR-15a/15b/16	I/R	CX3CL1	antifibrosis/anti-inflammation	[23]

I/R: ischemia/reperfusion; UUO: unilateral ureteral obstruction; Ang-II: Angiotensin-II; PKD: olycystic kidney disease; TGF-β1: transforming growth factor-β1; KD: knockdown; LPS: lipopolysaccharide; PTEN: phosphatase and tensin homolog deleted from chromosome 10; AKT: protein kinase B; mTOR: mammalian target of rapamycin; HIF: hypoxia-inducible factor; NF-κB: nuclear factor-κb; PRARα: peroxisome proliferator-activated receptorα; ERK: extracellular signal-regulated kinase; Sema3A: semaphorin3a; TLR4: toll-like receptor4; MMP9: matrix metalloproteinase9; *DRP1*: dynamic related protein 1; EMT: epithelial-mesenchymal transition; VEGF: vascular endothelial growth factor; BMP: bone morphogenetic protein; AhR: aryl hydrocarbon receptor; ACE: angiotensin-converting-enzyme; ROS: reactive oxygen species; PLA2R: phospholipase A2 receptor; CX3CL1: C-X-C motif chemokine ligand1; DKD: diabetic kidney disease.

## Abbreviations

MSCs	Mesenchymal stem cells
AKI	Acute kidney injury
CKD	Chronic kidney disease
EVs	Extracellular vesicles
miRNAs	MicroRNAs
ISEV	The International Society of Extracellular Vesicles
KEGG	The Kyoto Encyclopedia of Genes and Genomics
ECM	Extracellular matrix
PI3K	Phosphatidylinositol 3-kinase
AKT	Protein kinase B
PARTNER	Protein Analysis Through Evolutionary Relationships
HGF	Hepatocyte growth factor
IL	Interleukin
LIF	Leukemia inhibitory factor
CCL	C-C motif chemokine ligand
MCP-1	Monocyte chemotactic protein-1
VEGF	Vascular endothelial growth factor
CXCL	C-X-C motif chemokine ligand
TGF	Transforming growth factor
TSG	TNF-stimulated gene
NF-κB	Nuclear factor kappa-light-chain-enhancer of activated B cells
DAVID	The Database for Annotation, Visualization and Integrated Discovery
MAPK	Mitogen-activated protein kinase
PDGFR	Platelet-derived growth factor receptor
EGFR	Epidermal growth factor receptor
uPAR	Urokinase-type plasminogen activator receptor
PPAR	Peroxisome proliferator-activated receptor
BMP	Bone morphogenetic protein
PDGFC	Platelet-derived growth factor-C
ATG	Autophagy related gene
RT	Reverse transcription
PCR	Polymerase chain reaction
I/R	Ischemia/reperfusion
CX3CL	C-X3-C motif chemokine ligand
Sema3A	Semaphorin3a
ERK	Extracellular signal-regulated kinase
UUO	Unilateral ureteral obstruction
EMT	Epithelial-mesenchymal transition
MMP-9	Matrix metalloproteinase-9
ARF6	ADP-ribosylation factor 6
mTOR	Mammalian target of rapamycin
ATM	Ataxia telangiectasia-mutated
TNF	Tumor necrosis factor
P15	P15INK4b
P19	P19INK4d
PTEN	Phosphatase and tensin homologue deleted from chromosome 10
GSK	Glycogen synthase kinase
LPS	Lipopolysaccharide
ADPKD	Autosomal dominant polycystic kidney disease
TLR	Toll-like receptor
HIF	Hypoxia-inducible factor
Nrf2	NF-E2-related factor 2
AhR	Aryl hydrocarbon receptor
ROS	Reactive oxygen species
ACE	Angiotensin-converting-enzyme
PLA2R	Phospholipase A2 receptor
DM	Diabetes mellitus
OXSR1	Oxidative stress responsive 1
NOX	NADPH oxidase
PAN	Puromycin aminonucleoside
FGF	Fibroblast growth factor
HO-1	Heme oxygenase-1
NK	Natural killer
IDO	Indoleamine 2,3 dioxygenase
NO	Nitric oxide
PGE2	Prostaglandin E2
TCR	T cell receptor
Cox-2	Cyclooxygenase-2
IFN-γ	interferon-gamma
MHCI	major histocompatibility Class I
MHCII	major histocompatibility Class II
vWF	Von Willebrand factor
*DRP1*	Dynamic related protein 1
NRK-52E	Normal rat kidney-52E
MDA	Malondialdehyde
8-OhdG	8-hydroxy-2′-deoxyguanosine
